# Escore de Risco TIMI para Prevenção Secundária na Estratificação de Risco de Pacientes com Síndrome Coronariana Crônica: Estudo de Validação Externa

**DOI:** 10.36660/abc.20240821

**Published:** 2025-05-13

**Authors:** Henrique Trombini Pinesi, Eduardo Martelli Moreira, Marcelo Henrique Moreira Barbosa, Fabio Grunspun Pitta, Fabiana Hanna Rached, Eduardo Gomes Lima, Eduardo Bello Martins, Carlos Vicente Serrano

**Affiliations:** 1 Hospital das Clínicas Faculdade de Medicina Universidade de São Paulo São Paulo SP Brasil Instituto do Coração do Hospital das Clínicas da Faculdade de Medicina da Universidade de São Paulo, São Paulo, SP – Brasil; 2 Hospital Israelita Albert Einstein São Paulo SP Brasil Hospital Israelita Albert Einstein, São Paulo, SP – Brasil

**Keywords:** Doença Arterial Coronariana, Medição de Risco, Prevenção Secundária

## Abstract

**Fundamento:**

A estratificação de risco de pacientes com síndrome coronariana crônica (SCC) é desafiadora. O TIMI Risk Score for Secondary Prevention (TRS2P) é uma ferramenta simples de nove pontos desenvolvida para prever morte cardiovascular, infarto do miocárdio (IM) e acidente vascular cerebral isquêmico entre pacientes após IM. Nenhum estudo foi realizado sobre isso na população brasileira.

**Objetivo:**

Validar o TRS2P em pacientes com SCC em um centro terciário no Brasil.

**Métodos:**

Este é um estudo baseado em registro de pacientes com SCC, definido como tendo um procedimento de revascularização prévio, IM prévio ou estenose ≥50% em pelo menos uma artéria coronária epicárdica. O desfecho primário foi a incidência de eventos cardiovasculares maiores (MACE) em três anos (morte, IM ou acidente vascular cerebral). O risco predito foi conforme relatado no estudo de derivação original. A calibração foi avaliada por meio de um gráfico de calibração e do teste de Hosmer-Lemeshow. A discriminação foi avaliada por estatística de concordância (C). Um nível de significância de 0,05 foi adotado.

**Resultados:**

A amostra do estudo consistiu em 515 pacientes. Havia 173 (34%) mulheres, 75 (15%) tinham idade superior a 75 anos, 298 (58%) apresentavam diabetes e 156 (30%) doença renal crônica. Durante o acompanhamento, 126 MACE foram documentados. A incidência estimada em três anos foi de 24% [intervalo de confiança (IC) de 95% de 21%-28%], enquanto a incidência predita foi de 15%. Embora escores TRS2P mais altos estiveram associados a uma maior incidência de MACE, o modelo de escore de risco TRS2P subestimou a incidência de MACE em todos os estratos (p < 0,01). A estatística C foi de 0,64 (IC 95% 0,58-0,69).

**Conclusão:**

O escore TRS2P identificou pacientes com um risco mais alto de eventos cardiovasculares, mas subestimou MACE e apresentou baixa discriminação na coorte de paciente com SCC no Brasil.

## Graphical abstract

**Figura Central:**
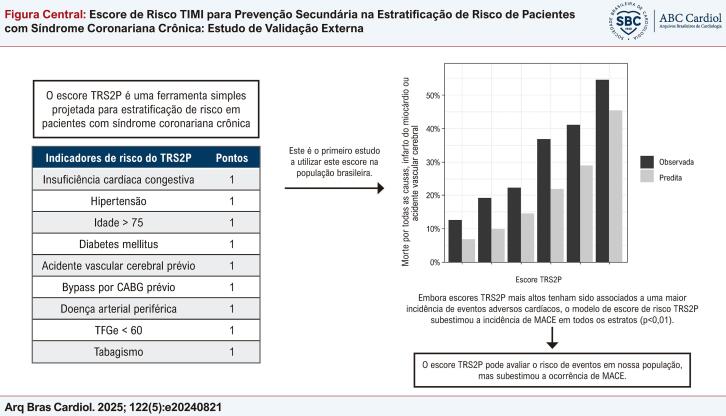
:Escore de Risco TIMI para Prevenção Secundária na Estratificação de Risco de Pacientes com Síndrome Coronariana Crônica: Estudo de Validação Externa CABG: coronary artery bypass graft surgery; MACE: major cardiovascular events; TFGe: taxa de filtração glomerular estimada.

## Introdução

A síndrome coronariana crônica (SCC) têm diferentes apresentações: pode apresentar um longo período estável, mas pode se tornar instável a qualquer momento, devido à síndrome coronariana aguda causada por ruptura ou erosão da placa. Esse processo dinâmico resulta em várias manifestações clínicas.^1^ Apesar dos avanços no tratamento farmacológico e nas estratégias de revascularização, permanece um risco de eventos cardiovasculares. A incidência desses eventos varia de acordo com vários fatores.^2^ Nesse cenário, a avaliação do risco é crítica para identificar os pacientes com risco maior para eventos cardiovasculares maiores (MACE, *Major Cardiovascular Events*) realocar recursos, acompanhar de perto os pacientes com risco aumento para eventos adversos, e otimizar o tratamento clínico desses pacientes.^3,4^

Um método de avaliação de risco é a utilização de escores clínicos para predizer o risco em longo prazo de MACE nos pacientes com SCC. Apesar de escores preditivos comumente usados, como o escore de risco de Framingham ou o escore SCORE (*Systematic Coronary Risk Evaluation*) Score terem sido desenvolvidos e validados em indivíduos sem doença cardiovascular, eles não foram validados em populações com SCC ou aterosclerose estabelecida.^5^ Há ainda uma necessidade de escores de risco validados na população brasileira.

O escore TIMI para prevenção secundária, ou TRS2P (*TIMI Risk Score for Secondary Prevention*) é um sistema de pontuação de risco simples baseado em nove variáveis clínicas (Tabela 1). Anteriormente, o TRS2P era usado para prever os desfechos cardiovasculares em três anos após um infarto do miocárdio (IM) recente em um grande ensaio clínico randomizado que testou o uso de Vorapaxar para prevenção secundária.^3^ O escore TRS2P também mostrou a capacidade de selecionar pacientes que se beneficiariam de uma intensificação da terapia para dislipidemia com Ezetimibe. Esse escore já foi validado em populações da América do Norte, Israel e alguns países europeus, mas não há dados de populações na América do Sul. Nosso objetivo foi validar o uso do escore TRS2P para avaliação de risco entre pacientes com SCC em um centro terciário no Brasil.^6,7^

**Tabela 1:** – Pontuação no escore TIMI Risk Score for Secondary Prevention (TRS2P)

Indicadores de risco do TRS2P	Pontos
Insuficiência cardíaca congestiva	1
Hipertensão	1
Idade > 75	1
Diabetes mellitus	1
Acidente vascular cerebral prévio	1
Bypass por CABG prévio	1
Doença arterial periférica	1
TFGe < 60	1
Tabagismo	1

CABG: coronary artery bypass graft surgery; TFGe: taxa de filtração glomerular estimada.

## Materiais e métodos

Este é um estudo do tipo coorte aninhado de acompanhamento, de um registro observacional prospectivo previamente publicado.^8^ Em resumo, entre janeiro de 2016 e maio de 2023, recrutamos pacientes com SCC estável que estavam sendo acompanhados em nosso ambulatório. Para serem considerados elegíveis, os pacientes deveriam ter um histórico de cirurgia de revascularização do miocárdio ou de intervenção coronariana percutânea, ou presença de lesões ≥ 50% na artéria coronária documentadas. Para este relatório, restringimos a amostra a pacientes em que o escore TRS2P poderia ser calculado, e que tinham um acompanhamento completo de três anos. Como apenas um paciente tinha um escore TRS2P de sete ou mais, ele também foi excluído devido a considerações estatísticas.

A coleta de dados foi prospetiva e padronizada. Os pacientes foram acompanhados anualmente de preferência, ou por telefone. Todos os pacientes assinaram um termo de consentimento. O desfecho foi a incidência de MACE, um composto de morte por todas as causas, IM não fatal ou acidente vascular cerebral (AVC) não fatal, em três anos. O escore original^3^ baseou-se em morte cardiovascular, no entanto, como os dados sobre a causa da morte não estavam disponíveis, optamos por usar a morte por todas as causas. Os eventos não foram revisados; confiamos em registros de saúde, bancos de dados governamentais e relatos de pacientes. Como nosso centro é uma referência para o tratamento de SCC, muitos desses pacientes foram tratados em nossas instalações. Os demais pacientes foram solicitados a trazer registros de saúde de outros provedores.

### Análise estatística

Os dados foram resumidos como porcentagens (%). O teste exato de Fisher foi usado para avaliar a associação univariada entre variáveis e o desfecho. A incidência de MACE observada em três anos foi estimada usando o método de Kaplan-Meier. As razões de risco (*hazard ratio*, HR) dos fatores prognósticos foram estimadas usando modelagem de riscos proporcionais de Cox. A incidência prevista do desfecho foi conforme relatada no estudo de derivação original. A calibração foi avaliada por um gráfico de calibração e do teste de Hosmer-Lemeshow. A discriminação foi avaliada por meio da curva Característica de Operação do Receptor (ROC) e da estatística de concordância (C). Valores de p < 0,05 foram considerados estatisticamente significativos. As análises foram realizadas com o programa R versão 4.3.1.^9^

## Resultados

**Figura 1:**
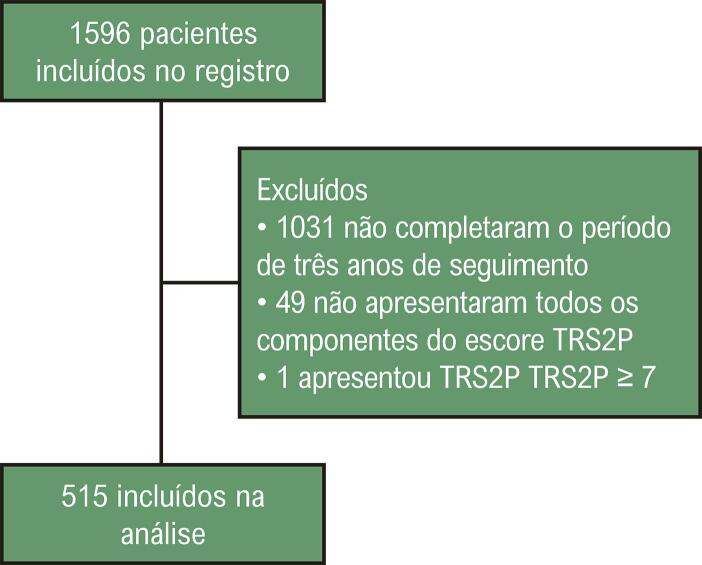
– Fluxograma do estudo.

Dos 1596 pacientes inscritos no registro, 515 foram incluídos na análise (Figura 1). As características basais e a incidência de cada componente do escore comparando a população com e sem um evento estão descritas na Tabela 2.

**Tabela 2:** – Características basais, componentes individuais do TIMI Risk Score for Secondary Prevention (TRS2P), e Eventos Cardiovasculares Maiores (MACE)

	Morte, infarto do miocárdio e AVC em três anos		Valor p^**1**^
	Sim, n = 126 n (%)	Não, n = 389 n (%)	
Mulheres	47 (37%)	126 (32%)	0,3
Idade > 75 anos	31 (25%)	44 (11%)	<0,001
Cirurgia de **bypass** da artéria coronária	49 (39%)	127 (33%)	0,2
Intervenção coronária percutânea	48 (38%)	187 (48%)	0,051
Infarto do miocárdio prévio	71 (56%)	250 (64%)	0,11
AVC prévio	11 (8,7%)	19 (4,9%)	0,13
Insuficiência cardíaca	38 (30%)	80 (21%)	0,029
Hipertensão	122 (97%)	374 (96%)	>0,9
Diabetes mellitus	80 (63%)	218 (56%)	0,15
Doença arterial periférica	11 (8,7%)	19 (4,9%)	0,13
Doença renal crônica	57 (45%)	99 (25%)	<0,001
Tabagismo atual	16 (13%)	51 (13%)	>0,9
Escore TRS2P			<0,001
0	0 (0%)	6 (1,5%)	
1	8 (6,3%)	56 (14%)	
2	29 (23%)	122 (31%)	
3	35 (28%)	122 (31%)	
4	32 (25%)	55 (14%)	
5	16 (13%)	23 (5,9%)	
6	6 (4,8%)	5 (1,3%)	

^1^ Teste exato de Fisher; AVC: acidente vascular cerebral.

**Figure 2:**
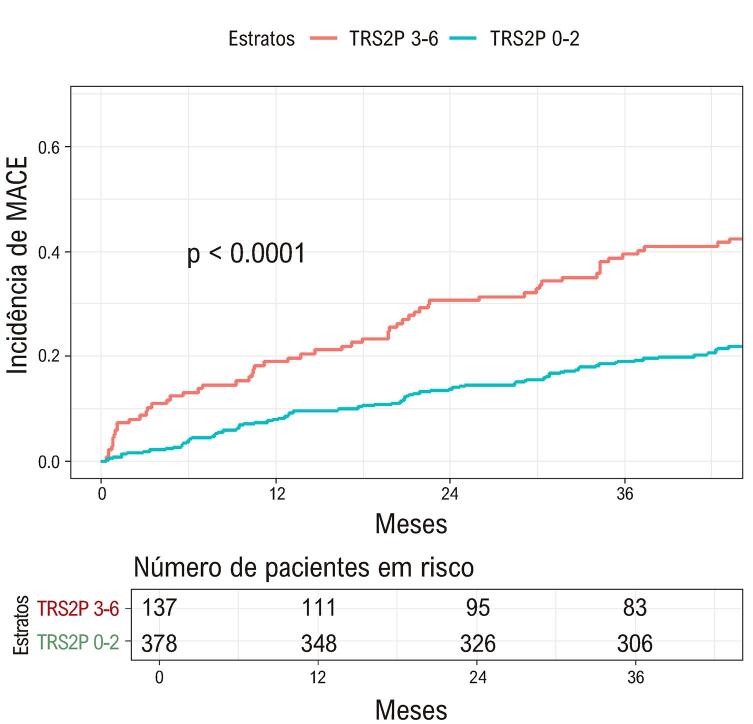
- Curva de Kaplan-Meyer mostrando a incidência de eventos cardiovasculares maiores (MACE) estratificada pelo escore TRS2 (TIMI Risk Score for Secondary Prevention).

Após três anos de acompanhamento, 126 MACEs foram documentados: 104 mortes, 14 IMs não fatais e oito acidentes vasculares cerebrais não fatais. Idade > 75 anos (p < 0,001), insuficiência cardíaca (p = 0,03), doença renal crônica (p < 0,001) e escore TRS2P (p < 0,001) foram associados a MACE na análise não ajustada (Figura 2). Um modelo de regressão de Cox de todos os componentes do escore mostrou que idade, doença renal crônica, tabagismo atual e cirurgia de *bypass* da artéria coronária (CABG) prévia foram independentemente associados a MACE, em ordem decrescente de razões de risco (Tabela 3).

**Tabela 3:** – Regressão de Cox dos componentes do TIMI Risk Score for Secondary Prevention (TRS2P)

Característica	HR	IC95%	Valor p
Insuficiência cardíaca	1,30	0,93, 1,84	0,13
Hipertensão	1,04	0,42, 2,56	>0,9
Idade > 75 anos	1,92	1,32, 2,80	<0,001
Diabetes mellitus	1,25	0,90, 1,73	0,2
AVC prévio	1,33	0,73, 2,42	0,4
Cirurgia de *bypass* da artéria coronária	1,41	1,04, 1,92	0,029
Doença arterial periférica	1,70	0,98, 2,95	0,059
Doença renal crônica	1,67	1,21, 2,30	0,002
Tabagismo atual	1,58	1,04, 2,40	0,033

HR: hazard ratio; IC: intervalo de confiança; AVC: acidente vascular cerebral.

**Figura 3:**
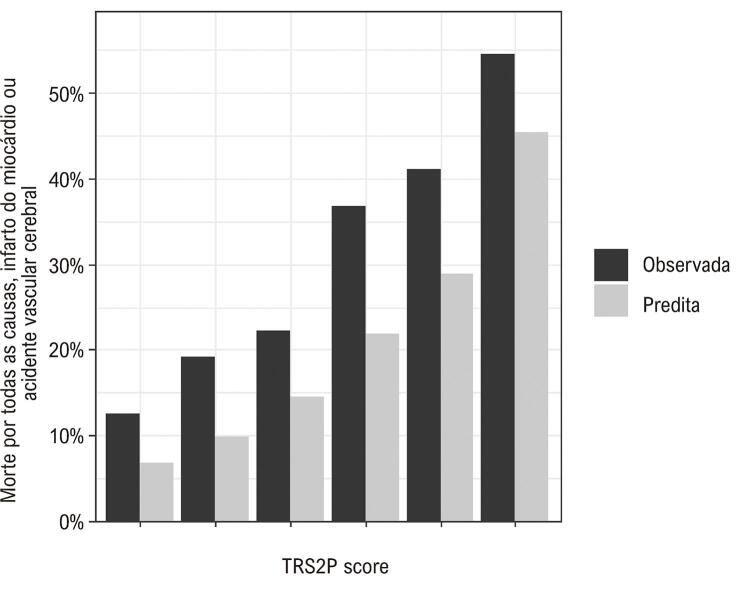
– Gráfico de calibração mostrando a incidência observada e a prevista de eventos cardiovasculares maiores em cada estrato do TRS2 (TIMI Risk Score for Secondary Prevention).

**Figura 4:**
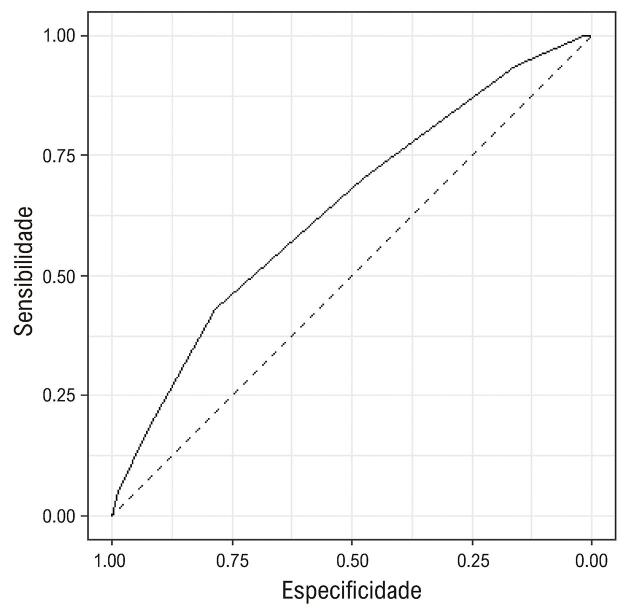
– Curva ROC mostrando a discriminação do TRS2 (TIMI Risk Score for Secondary Prevention) na população estudada. AUC: área sob a curva 0,64 (IC95%: 0,58-0,69).

A incidência estimada de MACE em três anos foi 24% (IC95% 21-28%), enquanto a incidência prevista foi de 15% (IC 95% 10-22%). Embora escores TRS2P mais altos estiveram associados a uma maior incidência de MACE, o modelo de escore de risco TRS2P subestimou a incidência de MACE em todas as estratificações (p < 0,01) (Figura 3). A Figura 4 mostra a curva ROC demonstrando a discriminação do TRS2P. A estatística C foi 0,64 (IC95%: 0,58-0,69).

## Discussão

Nosso estudo mostrou que o escore TRS2P pôde avaliar o risco de eventos em nossa população, mas subestimou MACE e apresentou discriminação moderada (Figura Central). A estratificação de risco em pacientes com SCC é desafiadora e muito importante. Nesse cenário, há uma falta de escores clínicos disponíveis. Portanto, modelos de predição de risco devem ser desenvolvidos e validados em diferentes populações. A avaliação precisa do risco pode levar a um cuidado mais eficaz ao paciente com a implementação adequada de intervenções preventivas.

Há uma grande variedade de escores de avaliação de risco validados para prevenção primária em síndromes coronarianas agudas.^10-13^ Por outro lado, os escores de risco para pacientes com SCC não são tão desenvolvidos. A estratificação de risco poderia oferecer aos médicos uma estratégia prática para identificar os pacientes que poderiam mais se beneficiar de terapia preventiva secundária intensiva, e também aqueles que se beneficiaram quanto aos custos, efeitos colaterais e polifarmácia.^2^ Isso é particularmente verdadeiro considerando as novas terapias para redução de risco e direcionamento de risco residual na SCC.^14,15^ O escore ideal deve ser prático, simples de usar e, de preferência, com variáveis clínicas disponíveis na prática diária. O escore SMART foi desenvolvido na população europeia para prever o risco de eventos cardiovasculares em 10 anos em pacientes com doença cardiovascular prévia.^5^ Este escore utiliza 11 variáveis e necessita de uma calculadora, enquanto o escore TRS2P é mais simples, usando somente nove variáveis de valor comparável.

Um dos motivos pelos quais o escore TRS2P tenha subestimado MACE neste artigo está ligado ao fato de que usamos morte por todas as causas, e não morte cardiovascular específica, como no artigo de derivação. Isso foi feito porque os dados sobre a causa da morte não estavam disponíveis. Segundo, usamos uma coorte da vida real, enquanto o escore foi derivado de um ensaio clínico randomizado. Populações de ensaios clínicos tendem a ser altamente selecionadas e podem representar indivíduos mais jovens e sadios. Os participantes de ensaios clínicos também podem ter acesso mais fácil aos cuidados de saúde, o que poderia contribuir a uma incidência de MACE mais baixa. Por exemplo, menos de 30% de nossos pacientes tinham um LDL-c de menos de 70 mg/dL durante o acompanhamento de dois anos.^8^ Cerca de um terço de nossos pacientes havia se submetido à cirurgia de CABG, em comparação a 13,6% no estudo original.^8^ Outra diferença chave é que o escore foi originalmente derivado de uma população com evento aterosclerótico recente (IM, AVC isquêmico ou doença arterial periférica sintomática). Em contraste, nosso estudo incluiu qualquer paciente com SCC (cerca de 60% tinham IM prévio em qualquer momento). Essas mesmas razões podem explicar a discriminação moderada.

Nosso estudo tem várias limitações que devem ser consideradas. Somente 32% de nossa coorte preencheram os mesmos critérios de inclusão do estudo original, o que pode introduzir um viés potencial. Além disso, nossa taxa de eventos é alta, refletindo a gravidade da doença em nossa população. Apesar disso, a incidência de IM é baixa, particularmente em comparação com as taxas de mortalidade. Essa discrepância pode ser atribuída a desafios na confirmação de MI, já que nosso registro depende de dados extraídos de registros médicos eletrônicos.

O escore TRS2P também foi testado em outras coortes da vida real.^7^ Um exemplo é o estudo conduzido por Williams et al.,^16^ que usou o escore em duas coortes após IM nos EUA, totalizando 9 618 pacientes, mostrando uma discriminação de risco consistente. Contudo, as taxas de eventos foram consistentemente mais altas nas coortes fora de ensaios.^16^ Zafrir et al.^6^ aplicaram o escore a 13 593 pacientes encaminhados para angiografia para avaliar ou tratar a doença coronariana em Israel, e encontraram também que o escore subestimou a incidência de MACE.^6^

Embora o escore TRS2P tenha subestimado a incidência de MACE, observamos uma correlação linear entre um escore mais alto e uma maior incidência de MACE, particularmente com um escore de três, mostrando que o escore é uma ferramenta potencial de rastreamento para pacientes que têm um risco residual mais alto e que poderiam se beneficiar da otimização do tratamento clínico, como alvos de LDL-c mais baixos, um tratamento antitrombótico mais intenso ou o uso de medicamentos anti-inflamatórios para reduzir o risco aterosclerótico residual. Nesse contexto, a adoção do TRS2P na prática clínica pode melhorar o tratamento de pacientes individuais com SCC, contribuindo para a redução na incidência de MACE.

## Conclusão

O escore TRS2P identifica pacientes com um risco maior de eventos cardiovasculares. No entanto, o escore subestimou a ocorrência de MACE e apresentou fraca discriminação entre os pacientes com SCC em um centro terciário no Brasil. Esses resultados demonstram os desafios da estratificação de risco na SCC e a necessidade de novas ferramentas para aprimorar a predição de risco.
