# Supplementary material for: Escore de Risco TIMI para Prevenção Secundária na Estratificação de Risco de Pacientes com Síndrome Coronariana Crônica: Estudo de Validação Externa
Source: Arq Bras Cardiol. 2025 May 13;122(5):e20240821. [Article in Portuguese] doi: 10.36660/abc.20240821 (PMC12129484; doi:10.36660/abc.20240821)
Supplement: Translation

# Translation: TIMI Risk Score for Secondary Prevention to Risk Stratify Chronic Coronary Syndrome Patients: External Validation Study

**DOI:** 10.36660/abc.20240821i

**Authors:** Henrique Trombini Pinesi, Eduardo Martelli Moreira, Marcelo Henrique Moreira Barbosa, Fabio Grunspun Pitta, Fabiana Hanna Rached, Eduardo Gomes Lima, Eduardo Bello Martins, Carlos Vicente Serrano

**Affiliations:** 1 Brazil Instituto do Coração do Hospital das Clínicas da Faculdade de Medicina da Universidade de São Paulo, São Paulo, SP – Brazil; 2 Brazil Hospital Israelita Albert Einstein, São Paulo, SP – Brazil

**Keywords:** Coronary Artery Disease, Risk Assessment, Secondary Prevention

## Abstract

**Background:**

Risk stratification in chronic coronary syndrome (CCS) patients is challenging. TIMI Risk Score for Secondary Prevention (TRS2P) is a simple nine-point tool developed to predict cardiovascular death, myocardial infarction (MI), and ischemic stroke among post-MI patients. No studies have been conducted on it in the Brazilian population.

**Objective:**

Validate the TRS2P score among CCS patients at a tertiary center in Brazil.

**Methods:**

This is a registry-based study of patients with CCS, defined as having a previous revascularization procedure, previous MI, or ≥50% stenosis in at least one epicardial coronary artery. The primary outcome was the three-year incidence of MACE (death, MI or stroke). The predicted risk was as reported in the original derivation study. Calibration was assessed through a calibration plot and the Hosmer-Lemeshow test. Discrimination was evaluated through the concordance (C)-statistic. A significance level of 0.05 was adopted.

**Results:**

The study sample consisted of 515 patients. There were 173 (34%) women, 75 (15%) aged over 75 years, 298 (58%) had diabetes, and 156 (30%) had chronic kidney disease. During follow-up, 126 MACE were documented. The estimated three-year incidence was 24% (95% confidence interval [CI] 21%-28%), whereas the predicted incidence was 15%. Although higher TRS2P scores were associated with higher MACE incidence, the TRS2P risk score model underestimated MACE incidence at every strata (p < 0.01). The C-statistic was 0.64 (95% CI 0.58-0.69).

**Conclusion:**

The TRS2P score identifies patients with a higher risk of cardiovascular events but it underestimated MACE and presented poor discrimination in a Brazilian CCS cohort.

## Introduction

Chronic coronary syndrome (CCS) can have different presentations: it can have a long stable period, but it can also become unstable at any time, due to acute coronary syndrome caused by plaque rupture or erosion. This dynamic process results in various clinical manifetations.^1^ Despite advances in pharmacological treatment and revascularization strategies, there remains a risk of cardiovascular events. The incidence of these events varies according to several factors.^2^ In this scenario, risk assessment is critical to identify the patients at higher risk of major cardiovascular events (MACE), relocate resources, closely follow patients with an elevated risk of adverse events, and optimize clinical treatment of these patients.^3,4^

One risk assessment method is applying clinical scores to predict the long-term risk of MACE in patients with CCS. Although commonly used prediction scores such as the Framingham Risk Score or the Systematic Coronary Risk Evaluation (SCORE) score have been developed and validated in individuals without cardiovascular disease, they have not been validated in populations with CCS and established atherosclerosis.^5^ There is also a need for validated risk scores in the Brazilian population.

The TIMI Risk Score for Secondary Prevention (TRS2P) is a simple risk-scoring system based on nine clinical variables (Table 1). Previously, TRS2P was used to predict three-year cardiovascular outcomes after a recent myocardial infarction (MI) in a large randomized clinical trial that tested the use of Vorapaxar for secondary prevention.^3^ The TRSP2 score also showed the ability to select patients with a net clinical benefit of intensifying the lipid target therapy with Ezetimibe.^4^ This score has already been validated in the North American and Israeli populations and in some European countries,^6,7^ but there are no data on the South American population. We aimed to validate the use of the TRSP2 score for risk assessment among patients with CCS at a tertiary center in Brazil.

**Table 1:** – TIMI Risk Score for Secondary Prevention (TRS2P) score points

TRS2P risk indicators	Points
Congestive Heart Failure	1
Hypertension	1
Age > 75	1
Diabetes mellitus	1
Prior stroke	1
Prior CABG	1
PAD	1
eGFR < 60	1
Smoking	1

CABG: coronary artery bypass graft surgery; PAD: peripheral artery disease; eGFR: estimated glomerular filtration rate.

## Material and methods

This is a nested cohort follow-up study in a previously published prospective observational registry.^8^ In brief, from January 2016 until May 2023 we enrolled patients with stable CCS that were being followed at our outpatient clinic. Patients must have had a history of coronary artery bypass surgery, percutaneous coronary intervention, or documented coronary artery lesions ≥ 50% to be eligible. For this report, we restricted the sample to patients in whom the TRS2P score could be calculated and had a complete three-year follow-up. As only one patient had a TRS2P score of seven or more, he was also excluded due to statistical considerations.

Data collection was standardized and prospective. Patients were followed yearly in person preferentially, or via phone contact otherwise. All patients provided signed consent. The endpoint was the incidence of MACE, a composite of all-cause death, nonfatal MI, or nonfatal stroke, at three years. The original score^3^ was derived based on cardiovascular death, however as the cause of death data was unavailable, we opted to use all-cause death instead. The events were not adjudicated; we relied on health records, governmental databases, and patient reports. Since our center is a referral center for the treatment of CCS, many of those patients were treated at our facilities. If not, the patients were asked to bring health records from other providers.

### Statistical analysis

Data was summarized as percentages (%). Fisher’s exact test was used to assess the univariate association between variables and the endpoint. The observed three-year MACE incidence was estimated using the Kaplan-Meier method. Hazard ratios (HR) of prognostic factors were estimated using Cox proportional hazards modeling. The predicted endpoint incidence was as reported in the original derivation study.^3^ Calibration was assessed through a calibration plot and the Hosmer-Lemeshow test. Discrimination was assessed through the Receiver Operating Characteristic (ROC) curve and concordance (C)-statistic. Values of p < 0.05 were considered statistically significant.

Analysis was conducted with R version 4.3.1.^9^

## Results

Out of the 1,596 currently enrolled patients in the registry, 515 were included in the analysis (Figure 1). The baseline characteristics and the incidence of each component of the score comparing the population with and without an event are described in Table 2.

**Figure 1:** – Flowchart of the study.

**Table 2:** – Baseline characteristics, individual Components of TIMI Risk Score for Secondary Prevention (TRS2P) and Major Cardiovascular Events (MACE)

	Death, myocardial infarction, or stroke at 3 years		p-value^**1**^
	Yes, n = 126 n (%)	No, N = 389 n (%)	
Female	47 (37%)	126 (32%)	0.3
Age > 75 years	31 (25%)	44 (11%)	<0.001
Coronary artery bypass graft surgery	49 (39%)	127 (33%)	0.2
Percutaneous coronary intervention	48 (38%)	187 (48%)	0.051
Previous myocardial infarction	71 (56%)	250 (64%)	0.11
Previous stroke	11 (8.7%)	19 (4.9%)	0.13
Heart failure	38 (30%)	80 (21%)	0.029
High blood pressure	122 (97%)	374 (96%)	>0.9
Diabetes mellitus	80 (63%)	218 (56%)	0.15
Peripheral artery disease	11 (8.7%)	19 (4.9%)	0.13
Chronic kidney disease	57 (45%)	99 (25%)	<0.001
Current smoking	16 (13%)	51 (13%)	>0.9
TRS2P score			<0.001
0	0 (0%)	6 (1.5%)	
1	8 (6.3%)	56 (14%)	
2	29 (23%)	122 (31%)	
3	35 (28%)	122 (31%)	
4	32 (25%)	55 (14%)	
5	16 (13%)	23 (5.9%)	
6	6 (4.8%)	5 (1.3%)	

^1^ Fisher’s exact test.

At three years of follow-up, 126 MACEs were documented: 104 deaths, 14 nonfatal MIs, and eight nonfatal strokes. Age > 75 years (p < 0.001), heart failure (p = 0.03), chronic kidney disease (p < 0.001), and TRS2P score (p < 0.001) were associated with MACE on unadjusted analysis (Figure 2). A Cox regression model with all score components showed that age, chronic kidney disease, current smoking and previous CABG were independently associated with MACE, in decreasing order of hazard ratios (Table 3).

**Figure 2:** – Kaplan-Meyer showing the incidence of major cardiovascular events (MACE) stratified by the TIMI Risk Score for Secondary Prevention (TRS2P).

**Table 3:** – Cox regression of the TIMI Risk Score for Secondary Prevention (TRS2P) score components

Characteristic	HR	95% CI	p-value
Heart failure	1.30	0.93, 1.84	0.13
High blood pressure	1.04	0.42, 2.56	>0.9
Age > 75 years	1.92	1.32, 2.80	<0.001
Diabetes mellitus	1.25	0.90, 1.73	0.2
Previous stroke	1.33	0.73, 2.42	0.4
Coronary artery bypass graft surgery	1.41	1.04, 1.92	0.029
Peripheral artery disease	1.70	0.98, 2.95	0.059
Chronic kidney disease	1.67	1.21, 2.30	0.002
Current smoking	1.58	1.04, 2.40	0.033

HR: hazard ratio; CI: confidence interval.

The estimated three-year incidence of MACE was 24% (95%CI 21-28%), whereas the predicted incidence was 15% (95% CI 10-22%). Although higher TRS2P scores were associated with higher MACE incidence, the TRS2P risk score model underestimated MACE incidence at every strata (p < 0.01) (Figure 3). Figure 4 shows the ROC curve demonstrating the discrimination of the TRS2P. The C-statistic was 0.64 (95%CI: 0.58-0.69).

**Figure 3:** – Calibration plot showing the observed and the predicted incidence of major cardiovascular events (MACE) in each TIMI Risk Score for Secondary Prevention (TRSP2) strata.

**Figure 4:** – ROC curve demonstrating the discrimination of the TIMI Risk Score for Secondary Prevention (TRS2P) in the study population. Area under the curve (AUC) 0.64 (95% CI: 0.58-0.69).

## Discussion

Our study showed that the TRS2P score could assess the risk of events in our population, but it underestimated MACE and presented moderate discrimination (Central Illustration). Risk stratification in CCS patients is challenging and very important. In this scenario, there is a lack of clinical scores available. Therefore, risk prediction models must be developed and validated in different populations. Accurate risk assessment may lead to more effective patient care with appropriate implementation of preventive interventions.

A wide variety of risk assessment scores are validated for primary prevention in acute coronary syndromes.^10-^ On the other hand, risk scores for CCS patients are not as developed. Risk stratification could offer physicians a practical strategy to identify those patients who would most benefit from intensive secondary preventive therapy, and also those who would benefit in terms of costs, side effects, and polypharmacy. This is particularly true considering the novel therapies for risk reduction and residual risk in CCS.^,15^ The ideal score must be practical, simple to use, and, preferably, with clinical variables available in the day-to-day practice. The SMART risk score was developed in the European population to predict the 10-year risk of cardiovascular events in patients with previous cardiovascular disease.^5^ This score uses 11 variables and needs a calculator, whereas the TRS2P score is simpler, using only nine equally valued variables.

One of the reasons why the TRS2P score underestimated MACE in this paper is linked to the fact that we used all-cause death, instead of cardiovascular specific as in the derivation paper. It was done so because the cause of death data was unavailable. Second, we used a real-life cohort, whereas the score was derived from a randomized clinical trial. Clinical trial populations tend to be highly selected and may represent younger and healthier individuals. Clinical trial participants may also have easier access to healthcare, which could contribute to a lower MACE incidence rate. For example, less than 30% of our patients had an LDL-c of less than 70 mg/dL during the two-year follow-up.^8^ About a third of our patients had undergone CABG surgery, compared to 13.6% in the original study.^8^ Another key difference is that the score was originally derived from a population with recent atherosclerotic event (MI, ischemic stroke, or symptomatic peripheral artery disease). In contrast, our study included any CCS patient (about 60% had previous MI at any time). These same reasons may account for the moderate discrimination.

Our study has several limitations that should be noted. Only 32% of our cohort met the same inclusion criteria as the original study, which may introduce potential bias. Additionally, our event rate is high, reflecting the severity of illness in our population. Despite this, the incidence of MI is low, particularly in comparison to mortality rates. This discrepancy could be attributed to challenges in confirming MI, as our registry relies on data extracted from electronic medical records.

The TRS2P score was also tested in other real-life cohorts.^7^ One example is the study conducted by Williams et al.,^16^ which used the score in two post-MI cohorts in the US, totaling 9,618 patients, showing a consistent risk discrimination. However, event rates were consistently higher in the non-trial cohorts.^16^ Zafrir et al.^6^ applied the score to 13,593 patients referred to angiography in Israel to assess or treat coronary disease and also found that the score underestimated MACE incidence.^6^

While the TRS2P score underestimated the incidence of MACE, we observed a linear correlation between a higher score and a higher MACE, particularly with a score of three, showing that the score is a potential screening tool for patients that have a higher residual risk and could benefit from clinical treatment optimization, such as lower LDL-c targets, a more intense antithrombotic or the use of anti-inflammatory drugs to reduce the residual atherosclerotic risk. In this context, the adoption of TRS2P in the clinical practice may enhance the treatment of individual patients with CCS, contributing to a reduction in the incidence of MACE.

## Conclusion

The TRS2P score identifies patients at a higher risk of cardiovascular events. However, it underestimated MACE and presented poor discrimination among patients with CCS at a tertiary center in Brazil. These results demonstrate the challenges of risk stratification in CCS and the need for novel tools to further enhance risk prediction.
